# {*N*,*N*-Bis[2-(diphenyl­phosphan­yl)eth­yl]­aniline}(η^2^-dibenzyl­ideneacetone)­palladium(0)

**DOI:** 10.1107/S1600536811033800

**Published:** 2011-09-14

**Authors:** Seyma Gören Keskin, Julie M. Stanley, Michelle L. Mejía, Bradley J. Holliday

**Affiliations:** a Department of Chemistry and Biochemistry, The University of Texas at Austin, 1 University Station, A5300, Austin, Texas 78712, USA

## Abstract

In the title complex, [Pd(C_34_H_33_NP_2_)(C_17_H_14_O)], the Pd^0^ atom is coordinated in a trigonal planar geometry formed by two P atoms of a bis­[(diphenyl­phosphino)eth­yl]aniline ligand and a C=C (η^2^) bond involving the C atoms that are in the α,β positions relative to the central ketone of the dibenzyl­ideneacetone ligand.

## Related literature

For general background and the potential applications of palladium complexes incorporating multidentate ligands, see: Blower *et al.* (1997 ▶); Michos *et al.* (1992 ▶); Kostas (2001 ▶); Lee *et al.* (2006 ▶); Hii *et al.* (1999 ▶). For similar structures, see: Retbøll *et al.* (2002 ▶); Goddard *et al.* (1995 ▶).
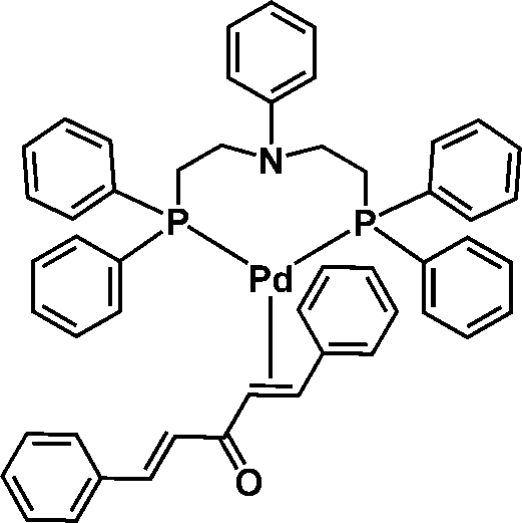


## Experimental

### 

#### Crystal data

[Pd(C_34_H_33_NP_2_)(C_17_H_14_O)]
*M* *_r_* = 858.24
Triclinic, 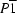

*a* = 10.087 (2) Å
*b* = 11.974 (2) Å
*c* = 17.473 (4) Å
α = 86.34 (3)°
β = 81.27 (2)°
γ = 83.15 (3)°
*V* = 2068.8 (7) Å^3^
*Z* = 2
Mo *K*α radiation
μ = 0.57 mm^−1^
*T* = 153 K
0.27 × 0.14 × 0.12 mm

#### Data collection

Nonius Kappa CCD diffractometer
Absorption correction: multi-scan (*DENZO* and *SCALEPACK*; Otwinowski & Minor, 1997 ▶) *T*_min_ = 0.837, *T*_max_ = 1.000
15976 measured reflections
9324 independent reflections
7745 reflections with *I* > 2σ(*I*)
*R*_int_ = 0.031

#### Refinement

*R*[*F* ^2^ > 2σ(*F* ^2^)] = 0.041
*wR*(*F* ^2^) = 0.089
*S* = 1.58
9324 reflections
505 parameters
H-atom parameters constrained
Δρ_max_ = 1.56 e Å^−3^
Δρ_min_ = −0.67 e Å^−3^

### 

Data collection: *COLLECT* (Nonius, 1998 ▶); cell refinement: *COLLECT*; data reduction: *DENZO* and *SCALEPACK* (Otwinowski & Minor, 1997 ▶); program(s) used to solve structure: *SIR97* (Altomare *et al.*, 1999 ▶) within *WinGX* (Farrugia, 1999 ▶); program(s) used to refine structure: *SHELXL97* (Sheldrick, 2008 ▶); molecular graphics: *ORTEP-3* (Farrugia, 1997 ▶); software used to prepare material for publication: *SHELXL97*.

## Supplementary Material

Crystal structure: contains datablock(s) I, global. DOI: 10.1107/S1600536811033800/lh5314sup1.cif

Structure factors: contains datablock(s) I. DOI: 10.1107/S1600536811033800/lh5314Isup2.hkl

Additional supplementary materials:  crystallographic information; 3D view; checkCIF report

## Figures and Tables

**Fig. 1.**
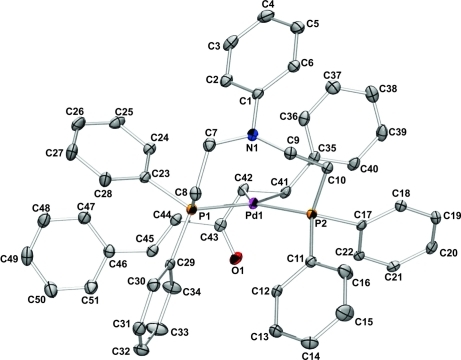
The molecular structure of dibenzylideneacetone {N,N-bis[(diphenylphosphino)ethyl]aniline}palladium(0) showing ellipsoids at the 30% probability level. Hydrogen atoms are omitted for clarity.

## supplementary crystallographic information

### Comment

Palladium complexes which incorporate multidentate ligands have been used in a variety of applications, such as catalysis, biotechnology and materials science (Blower *et al.*, 1997; Michos *et al.*, 1992; Kostas, 2001; Lee *et al.*, 2006). These multidentate ligands may contain donor atoms of the same type or be comprised of mixed donor atoms such as oxygen, carbon, phosphorous, sulfur and nitrogen (*i.e*., NNN, PNP, SPS). Advantages of mixed donor systems include flexible coordination modes and complex stability, both of which have the potential to increase performance in catalytic applications (Hii *et al.*, 1999), including coupling, hydrogenation and dehydrogenation reactions. Many examples of PNP-type (phosphorous/nitrogen/phosphorous) ligands have been studied because the hemilabile property of the nitrogen atom gives different coordination geometries, including tridentate monomeric (PNP), bidentate monomeric (PP) and bidentate dimeric (PP) modes, which can be controlled by substitution of the nitrogen atom, thereby affecting the nitrogen donor strength.

The molecular structure of the title compound is shown in Fig. 1. The geometry around the palladium atom is trigonal planar with the angle between the Pd—P1—P2 and Pd—C41—C42 planes being  1.40 °. The *N*,*N-*bis[(diphenylphosphino)-ethyl]aniline ligand is in a monomeric (PP) binding mode in which the nitrogen atom of the ligand is not bound to the metal center (distance between N1 and Pd1 is 3.405 Å). The average Pd1—P bond length is 2.326 Å, which is consistent with similar structures reported in the literature (Retbøll *et al.*, 2002; Goddard *et al.*, 1995). Dibenzylideneacetone (dba) is bound to Pd1 *via* one of the carbon-carbon double bonds in an η^2^ fashion, with the C41=C42 bond (1.411 (3)Å) slightly elongated due to complexation when compared to C44=C45 (1.327 (3) Å) and the C41=C42 centroid-Pd1 distance is 2.044 Å. Similar Pd(0) coordination environments have been previously reported with chelating diphosphine and dba ligands which also display the elongated carbon-carbon double bond (1.417 (3) Å) (Retbøll *et al.*, 2002). This coordination mode is not surprising since Pd_2_dba_3_ is the metal precursor used in the synthesis of the title complex and includes two palladium atoms with each metal bound η^2^ to the three dba ligands.

### Experimental

To 0.202 g of *N,N*-bis[(diphenylphosphino)ethyl]aniline under nitrogen in 5 ml anhydrous THF was added 0.179 g Pd_2_dba_3_. The reaction mixture was stirred at room temperature for 15 h, followed by filtration and removal of the solvent. Pure product was obtained by recrystallization from methylene chloride and hexanes yielding orange crystals suitable for diffraction. Purity and composition were confirmed by comparing ^1^H and ^31^P{^1^H} NMR spectroscopy and mass spectrometry data to literature values (Hii *et al.*, 1999). Yield = 54%.

### Refinement

All H atoms were positioned geometrically and refined using a riding model, with C—H = 0.95-0.99 Å and with *U*_iso_(H) = 1.2 times *U*_eq_(C).

### Crystal data

**Table:**

[Pd(C_34_H_33_NP_2_)(C_17_H_14_O)]	*Z* = 2
*M**_r_* = 858.24	*F*(000) = 888
Triclinic, *P*1	*D*_x_ = 1.378 Mg m^−^^3^
Hall symbol: -P 1	Melting point: 420 K
*a* = 10.087 (2) Å	Mo *K*α radiation, λ = 0.71073 Å
*b* = 11.974 (2) Å	Cell parameters from 27945 reflections
*c* = 17.473 (4) Å	θ = 1.0–27.5°
α = 86.34 (3)°	µ = 0.57 mm^−^^1^
β = 81.27 (2)°	*T* = 153 K
γ = 83.15 (3)°	Prism, orange
*V* = 2068.8 (7) Å^3^	0.27 × 0.14 × 0.12 mm

### Data collection

**Table:**

Nonius Kappa CCD diffractometer	9324 independent reflections
Radiation source: fine-focus sealed tube	7745 reflections with *I* > 2σ(*I*)
graphite	*R*_int_ = 0.031
ω–scans	θ_max_ = 27.5°, θ_min_ = 1.7°
Absorption correction: multi-scan (*DENZO* and *SCALEPACK*; Otwinowski & Minor, 1997)	*h* = −12→13
*T*_min_ = 0.837, *T*_max_ = 1.000	*k* = −14→15
15976 measured reflections	*l* = −22→22

### Refinement

**Table:**

Refinement on *F*^2^	Primary atom site location: structure-invariant direct methods
Least-squares matrix: full	Secondary atom site location: difference Fourier map
*R*[*F*^2^ > 2σ(*F*^2^)] = 0.041	Hydrogen site location: inferred from neighbouring sites
*wR*(*F*^2^) = 0.089	H-atom parameters constrained
*S* = 1.58	*w* = 1/[σ^2^(*F*_o_^2^) + (0.020*P*)^2^] where *P* = (*F*_o_^2^ + 2*F*_c_^2^)/3
9324 reflections	(Δ/σ)_max_ = 0.002
505 parameters	Δρ_max_ = 1.56 e Å^−^^3^
0 restraints	Δρ_min_ = −0.67 e Å^−^^3^

### Special details

**Table:**

Geometry. All e.s.d.'s (except the e.s.d. in the dihedral angle between two l.s. planes) are estimated using the full covariance matrix. The cell e.s.d.'s are taken into account individually in the estimation of e.s.d.'s in distances, angles and torsion angles; correlations between e.s.d.'s in cell parameters are only used when they are defined by crystal symmetry. An approximate (isotropic) treatment of cell e.s.d.'s is used for estimating e.s.d.'s involving l.s. planes.
Refinement. Refinement of *F*^2^ against ALL reflections. The weighted *R*-factor *wR* and goodness of fit *S* are based on *F*^2^, conventional *R*-factors *R* are based on *F*, with *F* set to zero for negative *F*^2^. The threshold expression of *F*^2^ > σ(*F*^2^) is used only for calculating *R*-factors(gt) *etc*. and is not relevant to the choice of reflections for refinement. *R*-factors based on *F*^2^ are statistically about twice as large as those based on *F*, and *R*- factors based on ALL data will be even larger.

### Fractional atomic coordinates and isotropic or equivalent isotropic displacement parameters (Å^2^)

**Table:**

	*x*	*y*	*z*	*U*_iso_*/*U*_eq_	
Pd1	0.699379 (17)	0.165949 (14)	0.714724 (10)	0.02103 (7)	
P1	0.76258 (6)	0.26802 (5)	0.60129 (3)	0.02080 (14)	
P2	0.88250 (6)	0.14942 (5)	0.78434 (3)	0.02179 (14)	
O1	0.60286 (17)	−0.08845 (14)	0.71914 (10)	0.0329 (4)	
N1	0.80565 (19)	0.41621 (16)	0.74653 (11)	0.0243 (5)	
C29	1.0050 (2)	0.0255 (2)	0.76262 (13)	0.0231 (5)	
C17	0.6584 (2)	0.26165 (19)	0.52443 (12)	0.0213 (5)	
C45	0.6737 (2)	−0.1747 (2)	0.86247 (14)	0.0283 (6)	
H45	0.6959	−0.2166	0.8170	0.034*	
C10	0.7601 (2)	0.42025 (19)	0.61030 (14)	0.0247 (5)	
H10A	0.7884	0.4555	0.5585	0.030*	
H10B	0.6665	0.4526	0.6285	0.030*	
C21	0.5339 (2)	0.3398 (2)	0.42294 (14)	0.0288 (6)	
H21	0.5012	0.4036	0.3933	0.035*	
C44	0.6082 (2)	−0.0729 (2)	0.85348 (14)	0.0267 (6)	
H44	0.5801	−0.0282	0.8973	0.032*	
C36	0.3912 (2)	0.3359 (2)	0.73626 (15)	0.0312 (6)	
H36	0.4375	0.3298	0.7801	0.037*	
C22	0.6075 (2)	0.3537 (2)	0.48183 (13)	0.0246 (5)	
H22	0.6231	0.4272	0.4930	0.030*	
C11	0.9295 (2)	0.22565 (19)	0.54657 (13)	0.0223 (5)	
C24	0.7150 (3)	0.1589 (2)	0.92741 (14)	0.0278 (6)	
H24	0.6434	0.1666	0.8972	0.033*	
C12	1.0002 (2)	0.1268 (2)	0.57025 (14)	0.0278 (6)	
H12	0.9654	0.0868	0.6162	0.033*	
C19	0.5585 (2)	0.1407 (2)	0.44922 (14)	0.0270 (6)	
H19	0.5423	0.0673	0.4380	0.032*	
C35	0.4079 (2)	0.2461 (2)	0.68703 (13)	0.0254 (6)	
C6	0.6271 (2)	0.5772 (2)	0.75423 (14)	0.0273 (6)	
H6	0.6659	0.6085	0.7059	0.033*	
C46	0.7162 (2)	−0.2308 (2)	0.93276 (14)	0.0295 (6)	
C23	0.8463 (2)	0.14303 (19)	0.89111 (13)	0.0251 (5)	
C40	0.3364 (2)	0.2593 (2)	0.62354 (14)	0.0315 (6)	
H40	0.3455	0.1998	0.5890	0.038*	
C20	0.5078 (2)	0.2336 (2)	0.40700 (14)	0.0291 (6)	
H20	0.4556	0.2245	0.3674	0.035*	
C47	0.6983 (3)	−0.1807 (2)	1.00444 (15)	0.0356 (6)	
H47	0.6571	−0.1054	1.0088	0.043*	
C14	1.1723 (3)	0.1405 (2)	0.46096 (16)	0.0351 (6)	
H14	1.2544	0.1109	0.4314	0.042*	
C30	1.1453 (2)	0.0263 (2)	0.75161 (14)	0.0297 (6)	
H30	1.1826	0.0938	0.7577	0.036*	
C32	1.1778 (3)	−0.1687 (2)	0.72396 (16)	0.0374 (7)	
H32	1.2361	−0.2351	0.7107	0.045*	
C31	1.2304 (3)	−0.0709 (2)	0.73177 (14)	0.0337 (6)	
H31	1.3254	−0.0691	0.7236	0.040*	
C38	0.2385 (3)	0.4442 (2)	0.65952 (16)	0.0386 (7)	
H38	0.1811	0.5111	0.6505	0.046*	
C18	0.6324 (2)	0.1547 (2)	0.50759 (13)	0.0238 (5)	
H18	0.6660	0.0907	0.5367	0.029*	
C51	0.7793 (3)	−0.3413 (2)	0.92972 (17)	0.0410 (7)	
H51	0.7931	−0.3775	0.8819	0.049*	
C9	0.8522 (2)	0.4495 (2)	0.66621 (13)	0.0266 (6)	
H9A	0.9442	0.4115	0.6507	0.032*	
H9B	0.8575	0.5317	0.6621	0.032*	
C5	0.5092 (3)	0.6314 (2)	0.79378 (16)	0.0368 (7)	
H5	0.4683	0.6992	0.7720	0.044*	
C48	0.7397 (3)	−0.2391 (3)	1.06887 (16)	0.0418 (7)	
H48	0.7252	−0.2040	1.1171	0.050*	
C2	0.6271 (3)	0.4334 (2)	0.85650 (14)	0.0297 (6)	
H2	0.6669	0.3653	0.8785	0.036*	
C42	0.5221 (2)	0.0900 (2)	0.76997 (14)	0.0253 (5)	
H42	0.5020	0.1320	0.8154	0.030*	
C16	0.9820 (3)	0.2829 (2)	0.47930 (16)	0.0403 (7)	
H16	0.9353	0.3513	0.4622	0.048*	
C43	0.5779 (2)	−0.0273 (2)	0.77551 (14)	0.0257 (5)	
C39	0.2532 (2)	0.3565 (2)	0.61000 (15)	0.0372 (7)	
H39	0.2059	0.3631	0.5666	0.045*	
C13	1.1214 (3)	0.0854 (2)	0.52758 (15)	0.0342 (6)	
H13	1.1696	0.0179	0.5450	0.041*	
C33	1.0403 (3)	−0.1711 (2)	0.7353 (2)	0.0509 (8)	
H33	1.0038	−0.2396	0.7312	0.061*	
C1	0.6888 (2)	0.4776 (2)	0.78474 (13)	0.0251 (5)	
C41	0.4974 (2)	0.1423 (2)	0.69805 (13)	0.0249 (5)	
H41	0.5428	0.1071	0.6527	0.030*	
C25	0.6851 (3)	0.1638 (2)	1.00788 (15)	0.0369 (7)	
H25	0.5939	0.1750	1.0321	0.044*	
C37	0.3084 (3)	0.4333 (2)	0.72226 (16)	0.0376 (7)	
H37	0.2995	0.4935	0.7562	0.045*	
C34	0.9547 (3)	−0.0739 (2)	0.75257 (17)	0.0391 (7)	
H34	0.8599	−0.0757	0.7576	0.047*	
C3	0.5102 (3)	0.4874 (2)	0.89517 (16)	0.0384 (7)	
H3	0.4697	0.4561	0.9431	0.046*	
C8	0.9909 (2)	0.26442 (19)	0.76920 (14)	0.0257 (5)	
H8A	1.0655	0.2465	0.8004	0.031*	
H8B	1.0313	0.2691	0.7140	0.031*	
C28	0.9503 (3)	0.1305 (2)	0.93644 (15)	0.0374 (7)	
H28	1.0414	0.1181	0.9123	0.045*	
C15	1.1036 (3)	0.2395 (2)	0.43694 (17)	0.0458 (7)	
H15	1.1394	0.2789	0.3909	0.055*	
C26	0.7889 (3)	0.1523 (2)	1.05210 (15)	0.0429 (7)	
H26	0.7691	0.1555	1.1069	0.052*	
C4	0.4513 (3)	0.5879 (3)	0.86398 (17)	0.0418 (7)	
H4	0.3716	0.6261	0.8910	0.050*	
C49	0.8019 (3)	−0.3480 (3)	1.06404 (17)	0.0470 (8)	
H49	0.8306	−0.3875	1.1086	0.056*	
C27	0.9215 (3)	0.1361 (3)	1.01663 (16)	0.0466 (8)	
H27	0.9928	0.1288	1.0470	0.056*	
C7	0.9154 (2)	0.3793 (2)	0.79127 (14)	0.0278 (6)	
H7A	0.8779	0.3754	0.8470	0.033*	
H7B	0.9804	0.4362	0.7835	0.033*	
C50	0.8220 (3)	−0.3990 (3)	0.99378 (18)	0.0480 (8)	
H50	0.8652	−0.4738	0.9898	0.058*	

### Atomic displacement parameters (Å^2^)

**Table:**

	*U*^11^	*U*^22^	*U*^33^	*U*^12^	*U*^13^	*U*^23^
Pd1	0.02037 (11)	0.02645 (12)	0.01626 (11)	−0.00308 (7)	−0.00279 (7)	0.00024 (8)
P1	0.0207 (3)	0.0243 (3)	0.0169 (3)	−0.0005 (3)	−0.0029 (3)	−0.0006 (3)
P2	0.0220 (3)	0.0262 (3)	0.0169 (3)	−0.0021 (3)	−0.0027 (3)	0.0001 (3)
O1	0.0411 (11)	0.0346 (10)	0.0253 (9)	−0.0077 (8)	−0.0074 (8)	−0.0081 (8)
N1	0.0286 (11)	0.0254 (11)	0.0192 (10)	−0.0001 (9)	−0.0063 (9)	−0.0012 (9)
C29	0.0251 (13)	0.0285 (13)	0.0142 (11)	0.0001 (10)	−0.0012 (10)	0.0011 (10)
C17	0.0196 (12)	0.0300 (13)	0.0130 (11)	−0.0008 (10)	0.0011 (9)	−0.0024 (10)
C45	0.0286 (14)	0.0335 (14)	0.0238 (13)	−0.0092 (11)	−0.0025 (11)	−0.0020 (12)
C10	0.0297 (13)	0.0235 (12)	0.0207 (12)	−0.0010 (10)	−0.0043 (11)	−0.0012 (11)
C21	0.0321 (14)	0.0298 (14)	0.0247 (13)	0.0024 (11)	−0.0105 (11)	0.0009 (11)
C44	0.0264 (13)	0.0326 (14)	0.0218 (13)	−0.0089 (11)	−0.0008 (11)	−0.0017 (11)
C36	0.0304 (14)	0.0399 (15)	0.0263 (14)	−0.0104 (12)	−0.0100 (11)	0.0015 (12)
C22	0.0255 (13)	0.0255 (13)	0.0224 (13)	−0.0009 (10)	−0.0038 (10)	−0.0010 (11)
C11	0.0207 (12)	0.0262 (13)	0.0199 (12)	−0.0024 (10)	−0.0024 (10)	−0.0020 (11)
C24	0.0321 (14)	0.0278 (14)	0.0238 (13)	−0.0062 (11)	−0.0028 (11)	−0.0026 (11)
C12	0.0330 (14)	0.0323 (14)	0.0167 (12)	0.0013 (11)	−0.0039 (11)	0.0004 (11)
C19	0.0296 (14)	0.0285 (14)	0.0233 (13)	−0.0056 (11)	−0.0001 (11)	−0.0077 (11)
C35	0.0184 (12)	0.0373 (15)	0.0212 (13)	−0.0088 (11)	−0.0010 (10)	−0.0003 (11)
C6	0.0307 (14)	0.0266 (13)	0.0268 (13)	−0.0025 (11)	−0.0099 (11)	−0.0061 (11)
C46	0.0264 (14)	0.0333 (15)	0.0304 (14)	−0.0056 (11)	−0.0074 (11)	−0.0009 (12)
C23	0.0320 (14)	0.0230 (13)	0.0200 (12)	−0.0022 (10)	−0.0032 (11)	−0.0011 (10)
C40	0.0218 (13)	0.0491 (17)	0.0231 (13)	−0.0031 (12)	−0.0015 (11)	−0.0046 (12)
C20	0.0271 (14)	0.0418 (16)	0.0191 (13)	−0.0054 (12)	−0.0041 (11)	−0.0029 (12)
C47	0.0289 (15)	0.0452 (16)	0.0321 (15)	0.0001 (12)	−0.0044 (12)	−0.0042 (13)
C14	0.0276 (14)	0.0355 (15)	0.0393 (16)	0.0011 (12)	0.0033 (12)	−0.0079 (14)
C30	0.0267 (14)	0.0354 (14)	0.0282 (14)	−0.0022 (11)	−0.0069 (11)	−0.0056 (12)
C32	0.0350 (16)	0.0336 (15)	0.0400 (16)	0.0063 (12)	−0.0005 (13)	−0.0055 (13)
C31	0.0224 (13)	0.0487 (17)	0.0292 (14)	0.0012 (12)	−0.0045 (11)	−0.0033 (13)
C38	0.0275 (15)	0.0439 (17)	0.0426 (17)	−0.0050 (12)	−0.0043 (13)	0.0129 (14)
C18	0.0252 (13)	0.0255 (13)	0.0185 (12)	0.0020 (10)	0.0004 (10)	−0.0014 (11)
C51	0.0532 (18)	0.0347 (16)	0.0390 (17)	−0.0075 (13)	−0.0161 (14)	−0.0035 (14)
C9	0.0298 (14)	0.0262 (13)	0.0242 (13)	−0.0014 (11)	−0.0063 (11)	−0.0009 (11)
C5	0.0410 (17)	0.0329 (15)	0.0388 (17)	0.0034 (12)	−0.0155 (14)	−0.0110 (13)
C48	0.0334 (16)	0.064 (2)	0.0289 (15)	−0.0039 (14)	−0.0077 (13)	−0.0063 (15)
C2	0.0357 (15)	0.0301 (14)	0.0251 (14)	−0.0070 (12)	−0.0060 (12)	−0.0045 (12)
C42	0.0239 (13)	0.0326 (14)	0.0216 (13)	−0.0094 (11)	−0.0050 (10)	−0.0032 (11)
C16	0.0399 (16)	0.0346 (15)	0.0378 (16)	0.0046 (13)	0.0091 (13)	0.0122 (13)
C43	0.0196 (12)	0.0339 (14)	0.0249 (13)	−0.0117 (11)	−0.0011 (10)	0.0004 (12)
C39	0.0235 (14)	0.0583 (19)	0.0297 (15)	−0.0045 (13)	−0.0076 (12)	0.0069 (14)
C13	0.0311 (15)	0.0373 (15)	0.0305 (15)	0.0088 (12)	−0.0027 (12)	−0.0010 (13)
C33	0.0401 (18)	0.0304 (16)	0.080 (2)	−0.0066 (13)	0.0052 (16)	−0.0119 (16)
C1	0.0273 (13)	0.0290 (13)	0.0206 (13)	−0.0033 (11)	−0.0064 (11)	−0.0066 (11)
C41	0.0189 (12)	0.0381 (15)	0.0179 (12)	−0.0067 (11)	−0.0003 (10)	−0.0032 (11)
C25	0.0453 (17)	0.0372 (16)	0.0255 (14)	−0.0087 (13)	0.0082 (13)	−0.0058 (13)
C37	0.0371 (16)	0.0351 (15)	0.0427 (17)	−0.0079 (13)	−0.0086 (13)	−0.0027 (13)
C34	0.0234 (14)	0.0345 (15)	0.0563 (19)	−0.0042 (12)	0.0062 (13)	−0.0050 (14)
C3	0.0414 (17)	0.0514 (18)	0.0252 (14)	−0.0131 (14)	−0.0038 (13)	−0.0112 (14)
C8	0.0250 (13)	0.0283 (13)	0.0246 (13)	−0.0048 (10)	−0.0067 (11)	0.0018 (11)
C28	0.0375 (16)	0.0485 (17)	0.0255 (14)	0.0040 (13)	−0.0094 (12)	−0.0016 (13)
C15	0.0420 (18)	0.0453 (18)	0.0413 (17)	−0.0059 (14)	0.0175 (14)	0.0097 (15)
C26	0.063 (2)	0.0445 (17)	0.0183 (14)	0.0002 (15)	0.0008 (14)	−0.0026 (13)
C4	0.0363 (16)	0.0512 (19)	0.0389 (17)	0.0008 (14)	−0.0047 (14)	−0.0221 (16)
C49	0.0449 (18)	0.062 (2)	0.0375 (17)	−0.0124 (16)	−0.0170 (14)	0.0135 (16)
C27	0.058 (2)	0.0569 (19)	0.0270 (15)	0.0048 (16)	−0.0193 (15)	−0.0033 (14)
C7	0.0324 (14)	0.0273 (13)	0.0262 (13)	−0.0066 (11)	−0.0091 (11)	−0.0023 (11)
C50	0.057 (2)	0.0389 (17)	0.052 (2)	−0.0024 (14)	−0.0248 (16)	0.0026 (15)

### Geometric parameters (Å, °)

**Table:**

Pd1—C41	2.155 (2)	C14—C13	1.362 (4)
Pd1—C42	2.170 (2)	C14—C15	1.375 (4)
Pd1—P1	2.3068 (10)	C14—H14	0.9500
Pd1—P2	2.3441 (9)	C30—C31	1.390 (3)
P1—C10	1.836 (2)	C30—H30	0.9500
P1—C17	1.837 (2)	C32—C31	1.366 (4)
P1—C11	1.837 (2)	C32—C33	1.374 (4)
P2—C29	1.835 (2)	C32—H32	0.9500
P2—C8	1.840 (2)	C31—H31	0.9500
P2—C23	1.844 (2)	C38—C37	1.382 (4)
O1—C43	1.242 (3)	C38—C39	1.384 (4)
N1—C1	1.411 (3)	C38—H38	0.9500
N1—C9	1.457 (3)	C18—H18	0.9500
N1—C7	1.460 (3)	C51—C50	1.376 (4)
C29—C34	1.379 (3)	C51—H51	0.9500
C29—C30	1.399 (3)	C9—H9A	0.9900
C17—C22	1.381 (3)	C9—H9B	0.9900
C17—C18	1.395 (3)	C5—C4	1.375 (4)
C45—C44	1.327 (3)	C5—H5	0.9500
C45—C46	1.459 (3)	C48—C49	1.379 (4)
C45—H45	0.9500	C48—H48	0.9500
C10—C9	1.529 (3)	C2—C3	1.377 (4)
C10—H10A	0.9900	C2—C1	1.413 (4)
C10—H10B	0.9900	C2—H2	0.9500
C21—C20	1.382 (3)	C42—C41	1.411 (3)
C21—C22	1.386 (3)	C42—C43	1.453 (3)
C21—H21	0.9500	C42—H42	0.9500
C44—C43	1.496 (3)	C16—C15	1.394 (4)
C44—H44	0.9500	C16—H16	0.9500
C36—C37	1.382 (4)	C39—H39	0.9500
C36—C35	1.398 (3)	C13—H13	0.9500
C36—H36	0.9500	C33—C34	1.384 (4)
C22—H22	0.9500	C33—H33	0.9500
C11—C12	1.380 (3)	C41—H41	0.9500
C11—C16	1.388 (4)	C25—C26	1.382 (4)
C24—C23	1.376 (3)	C25—H25	0.9500
C24—C25	1.396 (3)	C37—H37	0.9500
C24—H24	0.9500	C34—H34	0.9500
C12—C13	1.387 (3)	C3—C4	1.394 (4)
C12—H12	0.9500	C3—H3	0.9500
C19—C18	1.381 (3)	C8—C7	1.533 (3)
C19—C20	1.384 (3)	C8—H8A	0.9900
C19—H19	0.9500	C8—H8B	0.9900
C35—C40	1.403 (3)	C28—C27	1.391 (4)
C35—C41	1.467 (3)	C28—H28	0.9500
C6—C1	1.392 (3)	C15—H15	0.9500
C6—C5	1.392 (3)	C26—C27	1.383 (4)
C6—H6	0.9500	C26—H26	0.9500
C46—C51	1.399 (4)	C4—H4	0.9500
C46—C47	1.401 (3)	C49—C50	1.383 (4)
C23—C28	1.396 (3)	C49—H49	0.9500
C40—C39	1.380 (4)	C27—H27	0.9500
C40—H40	0.9500	C7—H7A	0.9900
C20—H20	0.9500	C7—H7B	0.9900
C47—C48	1.379 (4)	C50—H50	0.9500
C47—H47	0.9500		
			
C41—Pd1—C42	38.09 (9)	C19—C18—C17	120.9 (2)
C41—Pd1—P1	99.11 (7)	C19—C18—H18	119.5
C42—Pd1—P1	137.21 (7)	C17—C18—H18	119.5
C41—Pd1—P2	154.31 (7)	C50—C51—C46	122.1 (3)
C42—Pd1—P2	116.24 (7)	C50—C51—H51	119.0
P1—Pd1—P2	106.54 (3)	C46—C51—H51	119.0
C10—P1—C17	102.49 (11)	N1—C9—C10	112.9 (2)
C10—P1—C11	104.10 (11)	N1—C9—H9A	109.0
C17—P1—C11	99.22 (10)	C10—C9—H9A	109.0
C10—P1—Pd1	115.73 (8)	N1—C9—H9B	109.0
C17—P1—Pd1	115.60 (8)	C10—C9—H9B	109.0
C11—P1—Pd1	117.32 (8)	H9A—C9—H9B	107.8
C29—P2—C8	102.03 (11)	C4—C5—C6	120.8 (3)
C29—P2—C23	103.60 (11)	C4—C5—H5	119.6
C8—P2—C23	99.81 (11)	C6—C5—H5	119.6
C29—P2—Pd1	114.30 (8)	C49—C48—C47	120.8 (3)
C8—P2—Pd1	116.74 (8)	C49—C48—H48	119.6
C23—P2—Pd1	117.99 (8)	C47—C48—H48	119.6
C1—N1—C9	117.92 (19)	C3—C2—C1	121.2 (3)
C1—N1—C7	117.68 (18)	C3—C2—H2	119.4
C9—N1—C7	113.44 (19)	C1—C2—H2	119.4
C34—C29—C30	117.9 (2)	C41—C42—C43	120.9 (2)
C34—C29—P2	117.35 (18)	C41—C42—Pd1	70.40 (14)
C30—C29—P2	124.61 (18)	C43—C42—Pd1	100.38 (15)
C22—C17—C18	118.5 (2)	C41—C42—H42	119.5
C22—C17—P1	124.97 (17)	C43—C42—H42	119.5
C18—C17—P1	116.49 (17)	Pd1—C42—H42	99.1
C44—C45—C46	128.7 (2)	C11—C16—C15	119.8 (3)
C44—C45—H45	115.7	C11—C16—H16	120.1
C46—C45—H45	115.7	C15—C16—H16	120.1
C9—C10—P1	113.19 (17)	O1—C43—C42	123.2 (2)
C9—C10—H10A	108.9	O1—C43—C44	120.1 (2)
P1—C10—H10A	108.9	C42—C43—C44	116.7 (2)
C9—C10—H10B	108.9	C40—C39—C38	120.2 (2)
P1—C10—H10B	108.9	C40—C39—H39	119.9
H10A—C10—H10B	107.8	C38—C39—H39	119.9
C20—C21—C22	120.4 (2)	C14—C13—C12	120.7 (2)
C20—C21—H21	119.8	C14—C13—H13	119.6
C22—C21—H21	119.8	C12—C13—H13	119.6
C45—C44—C43	121.1 (2)	C32—C33—C34	120.3 (3)
C45—C44—H44	119.5	C32—C33—H33	119.9
C43—C44—H44	119.5	C34—C33—H33	119.9
C37—C36—C35	121.1 (2)	C6—C1—N1	123.7 (2)
C37—C36—H36	119.5	C6—C1—C2	117.7 (2)
C35—C36—H36	119.5	N1—C1—C2	118.5 (2)
C17—C22—C21	120.6 (2)	C42—C41—C35	125.9 (2)
C17—C22—H22	119.7	C42—C41—Pd1	71.51 (13)
C21—C22—H22	119.7	C35—C41—Pd1	115.32 (16)
C12—C11—C16	118.8 (2)	C42—C41—H41	117.0
C12—C11—P1	117.87 (19)	C35—C41—H41	117.0
C16—C11—P1	123.10 (19)	Pd1—C41—H41	83.0
C23—C24—C25	121.2 (2)	C26—C25—C24	119.6 (3)
C23—C24—H24	119.4	C26—C25—H25	120.2
C25—C24—H24	119.4	C24—C25—H25	120.2
C11—C12—C13	120.6 (2)	C36—C37—C38	120.9 (3)
C11—C12—H12	119.7	C36—C37—H37	119.6
C13—C12—H12	119.7	C38—C37—H37	119.6
C18—C19—C20	120.0 (2)	C29—C34—C33	121.0 (2)
C18—C19—H19	120.0	C29—C34—H34	119.5
C20—C19—H19	120.0	C33—C34—H34	119.5
C36—C35—C40	117.1 (2)	C2—C3—C4	120.1 (3)
C36—C35—C41	123.2 (2)	C2—C3—H3	120.0
C40—C35—C41	119.7 (2)	C4—C3—H3	120.0
C1—C6—C5	120.8 (2)	C7—C8—P2	113.37 (17)
C1—C6—H6	119.6	C7—C8—H8A	108.9
C5—C6—H6	119.6	P2—C8—H8A	108.9
C51—C46—C47	116.9 (2)	C7—C8—H8B	108.9
C51—C46—C45	119.1 (2)	P2—C8—H8B	108.9
C47—C46—C45	124.1 (2)	H8A—C8—H8B	107.7
C24—C23—C28	118.7 (2)	C27—C28—C23	120.5 (3)
C24—C23—P2	119.91 (18)	C27—C28—H28	119.7
C28—C23—P2	121.23 (19)	C23—C28—H28	119.7
C39—C40—C35	121.7 (2)	C14—C15—C16	120.8 (3)
C39—C40—H40	119.2	C14—C15—H15	119.6
C35—C40—H40	119.2	C16—C15—H15	119.6
C21—C20—C19	119.4 (2)	C25—C26—C27	120.0 (2)
C21—C20—H20	120.3	C25—C26—H26	120.0
C19—C20—H20	120.3	C27—C26—H26	120.0
C48—C47—C46	121.1 (2)	C5—C4—C3	119.5 (3)
C48—C47—H47	119.5	C5—C4—H4	120.2
C46—C47—H47	119.5	C3—C4—H4	120.2
C13—C14—C15	119.3 (2)	C48—C49—C50	119.4 (3)
C13—C14—H14	120.3	C48—C49—H49	120.3
C15—C14—H14	120.3	C50—C49—H49	120.3
C31—C30—C29	120.6 (2)	C26—C27—C28	120.0 (3)
C31—C30—H30	119.7	C26—C27—H27	120.0
C29—C30—H30	119.7	C28—C27—H27	120.0
C31—C32—C33	119.9 (2)	N1—C7—C8	113.58 (18)
C31—C32—H32	120.0	N1—C7—H7A	108.9
C33—C32—H32	120.0	C8—C7—H7A	108.9
C32—C31—C30	120.2 (2)	N1—C7—H7B	108.9
C32—C31—H31	119.9	C8—C7—H7B	108.9
C30—C31—H31	119.9	H7A—C7—H7B	107.7
C37—C38—C39	119.1 (3)	C51—C50—C49	119.8 (3)
C37—C38—H38	120.4	C51—C50—H50	120.1
C39—C38—H38	120.4	C49—C50—H50	120.1
			
C41—Pd1—P1—C10	102.55 (11)	C47—C46—C51—C50	0.1 (4)
C42—Pd1—P1—C10	102.35 (13)	C45—C46—C51—C50	179.9 (3)
P2—Pd1—P1—C10	−76.02 (9)	C1—N1—C9—C10	−71.2 (3)
C41—Pd1—P1—C17	−17.26 (10)	C7—N1—C9—C10	145.40 (19)
C42—Pd1—P1—C17	−17.46 (12)	P1—C10—C9—N1	−67.7 (2)
P2—Pd1—P1—C17	164.17 (8)	C1—C6—C5—C4	0.2 (4)
C41—Pd1—P1—C11	−133.85 (10)	C46—C47—C48—C49	1.1 (4)
C42—Pd1—P1—C11	−134.05 (12)	P1—Pd1—C42—C41	0.32 (18)
P2—Pd1—P1—C11	47.59 (8)	P2—Pd1—C42—C41	178.58 (11)
C41—Pd1—P2—C29	93.86 (17)	C41—Pd1—C42—C43	119.3 (2)
C42—Pd1—P2—C29	91.83 (11)	P1—Pd1—C42—C43	119.61 (14)
P1—Pd1—P2—C29	−89.40 (9)	P2—Pd1—C42—C43	−62.14 (15)
C41—Pd1—P2—C8	−147.22 (16)	C12—C11—C16—C15	0.4 (4)
C42—Pd1—P2—C8	−149.25 (11)	P1—C11—C16—C15	−173.9 (2)
P1—Pd1—P2—C8	29.51 (9)	C41—C42—C43—O1	−2.2 (4)
C41—Pd1—P2—C23	−28.29 (17)	Pd1—C42—C43—O1	−75.5 (2)
C42—Pd1—P2—C23	−30.32 (11)	C41—C42—C43—C44	176.4 (2)
P1—Pd1—P2—C23	148.44 (9)	Pd1—C42—C43—C44	103.1 (2)
C8—P2—C29—C34	−166.8 (2)	C45—C44—C43—O1	6.6 (4)
C23—P2—C29—C34	89.9 (2)	C45—C44—C43—C42	−172.0 (2)
Pd1—P2—C29—C34	−39.8 (2)	C35—C40—C39—C38	0.0 (4)
C8—P2—C29—C30	9.7 (2)	C37—C38—C39—C40	0.2 (4)
C23—P2—C29—C30	−93.6 (2)	C15—C14—C13—C12	1.4 (4)
Pd1—P2—C29—C30	136.65 (19)	C11—C12—C13—C14	−0.9 (4)
C10—P1—C17—C22	6.0 (2)	C31—C32—C33—C34	1.7 (5)
C11—P1—C17—C22	−100.8 (2)	C5—C6—C1—N1	177.7 (2)
Pd1—P1—C17—C22	132.78 (18)	C5—C6—C1—C2	0.5 (3)
C10—P1—C17—C18	−175.49 (17)	C9—N1—C1—C6	−9.6 (3)
C11—P1—C17—C18	77.73 (19)	C7—N1—C1—C6	132.3 (2)
Pd1—P1—C17—C18	−48.67 (19)	C9—N1—C1—C2	167.6 (2)
C17—P1—C10—C9	−174.41 (17)	C7—N1—C1—C2	−50.6 (3)
C11—P1—C10—C9	−71.42 (19)	C3—C2—C1—C6	−0.3 (3)
Pd1—P1—C10—C9	58.86 (19)	C3—C2—C1—N1	−177.7 (2)
C46—C45—C44—C43	177.5 (2)	C43—C42—C41—C35	161.1 (2)
C18—C17—C22—C21	−0.9 (3)	Pd1—C42—C41—C35	−108.3 (2)
P1—C17—C22—C21	177.59 (18)	C43—C42—C41—Pd1	−90.6 (2)
C20—C21—C22—C17	1.4 (4)	C36—C35—C41—C42	34.9 (4)
C10—P1—C11—C12	137.82 (18)	C40—C35—C41—C42	−146.5 (2)
C17—P1—C11—C12	−116.71 (19)	C36—C35—C41—Pd1	−49.9 (3)
Pd1—P1—C11—C12	8.5 (2)	C40—C35—C41—Pd1	128.7 (2)
C10—P1—C11—C16	−47.8 (2)	P1—Pd1—C41—C42	−179.78 (12)
C17—P1—C11—C16	57.6 (2)	P2—Pd1—C41—C42	−2.9 (2)
Pd1—P1—C11—C16	−177.17 (18)	C42—Pd1—C41—C35	121.7 (2)
C16—C11—C12—C13	0.0 (3)	P1—Pd1—C41—C35	−58.05 (17)
P1—C11—C12—C13	174.56 (18)	P2—Pd1—C41—C35	118.79 (18)
C37—C36—C35—C40	−0.4 (4)	C23—C24—C25—C26	−0.2 (4)
C37—C36—C35—C41	178.2 (2)	C35—C36—C37—C38	0.7 (4)
C44—C45—C46—C51	177.1 (3)	C39—C38—C37—C36	−0.6 (4)
C44—C45—C46—C47	−3.1 (4)	C30—C29—C34—C33	2.6 (4)
C25—C24—C23—C28	0.7 (4)	P2—C29—C34—C33	179.3 (2)
C25—C24—C23—P2	−174.62 (19)	C32—C33—C34—C29	−3.2 (5)
C29—P2—C23—C24	−133.7 (2)	C1—C2—C3—C4	−0.5 (4)
C8—P2—C23—C24	121.2 (2)	C29—P2—C8—C7	−174.39 (16)
Pd1—P2—C23—C24	−6.3 (2)	C23—P2—C8—C7	−68.07 (18)
C29—P2—C23—C28	51.0 (2)	Pd1—P2—C8—C7	60.27 (18)
C8—P2—C23—C28	−54.0 (2)	C24—C23—C28—C27	−1.2 (4)
Pd1—P2—C23—C28	178.47 (18)	P2—C23—C28—C27	174.1 (2)
C36—C35—C40—C39	0.0 (4)	C13—C14—C15—C16	−1.0 (4)
C41—C35—C40—C39	−178.7 (2)	C11—C16—C15—C14	0.1 (4)
C22—C21—C20—C19	−1.5 (4)	C24—C25—C26—C27	0.1 (4)
C18—C19—C20—C21	1.1 (4)	C6—C5—C4—C3	−1.1 (4)
C51—C46—C47—C48	−0.9 (4)	C2—C3—C4—C5	1.3 (4)
C45—C46—C47—C48	179.3 (2)	C47—C48—C49—C50	−0.4 (4)
C34—C29—C30—C31	−0.5 (4)	C25—C26—C27—C28	−0.5 (4)
P2—C29—C30—C31	−176.98 (19)	C23—C28—C27—C26	1.1 (4)
C33—C32—C31—C30	0.4 (4)	C1—N1—C7—C8	144.8 (2)
C29—C30—C31—C32	−1.0 (4)	C9—N1—C7—C8	−71.7 (3)
C20—C19—C18—C17	−0.7 (3)	P2—C8—C7—N1	−60.7 (2)
C22—C17—C18—C19	0.6 (3)	C46—C51—C50—C49	0.5 (5)
P1—C17—C18—C19	−178.07 (18)	C48—C49—C50—C51	−0.4 (5)
